# An Eddy Current-Based Structural Health Monitoring Technique for Tracking Bolt Cracking

**DOI:** 10.3390/s20236843

**Published:** 2020-11-30

**Authors:** Hu Sun, Tao Wang, Dawei Lin, Yishou Wang, Xinlin Qing

**Affiliations:** School of Aerospace Engineering, Xiamen University, Xiamen 361005, China; sunhu@xmu.edu.cn (H.S.); wangtao@stu.xmu.edu.cn (T.W.); a2234467@163.com (D.L.); wangys@xmu.edu.cn (Y.W.)

**Keywords:** bolt cracking, eddy current, sensing film, bolted joints, structural health monitoring

## Abstract

Bolted joints are the primary structures for the load transfer of large-scale structures. It is vital to monitor the process of bolt cracking for enduring structural safety. In this paper, a structural health monitoring technique based on the embedding eddy current sensing film has been proposed to quantify the crack parameters of bolt cracking. Two configurations of the sensing film containing one-dimensional circumferential coil array and two-dimensional coil array are designed and verified to have the ability to identify three crack parameters: the crack angle, the crack depth, and the crack location in the axial direction of the bolt. The finite element method has been employed not only to verify the capacity of the sensing film, but also to investigate the interaction between the crack and the eddy current/magnetic field. It has been demonstrated that as the crack propagates, the variations of the induced voltage of the sensing coils are influenced by both eddy current effect and magnetic flux leakage, which play different roles in the different periods of the crack propagation. Experiments have been performed to verify the effectiveness and feasibility of the sensing film to quantify three crack parameters in the process of the bolt cracking.

## 1. Introduction

Bolted joint is one of the most critical processes of assembly connection for large-scale structures, such as aircraft, civil engineering, train, marine structures, etc., because it plays a vital role in transferring the main load and ensuring the structural safety. However, the bolt cracking often occurs due to the electrochemical corrosion, stress corrosion, hydrogen embrittlement, fatigue, etc., which causes structural failure and has been a severe threat to the structural security [1,2,3]. Especially, the bolt is often used in the harsh environment such as complex stress, high temperature and high pressure, periodic vibration, etc., and is affected by temperature, stress, and vibration fatigue for a long time, which causes the initiation and progress of fatigue cracks. If the bolt crack cannot be detected in time, it can easily cause bolt fracture and more severe accidents.

Some nondestructive testing (NDT) methods are used to detect cracks in bolts. The ultrasonic testing method is the most commonly used NDT method. There are two classical methods used to detect cracks at the bolt: longitudinal wave method and shear wave method. Because of their different principles, the noticeable difference between the two methods is that the transducer of longitudinal wave is located at the top of the bolt, while that of the shear wave is located at the cylinder side of the bolt. Ultrasonic phased array technology is also used to visually scan and image the cracks at the bolt. Magnetic particle testing is also used to detect cracks in bolts. The magnetic flux leakage generated at the cracks on the surface of ferromagnetic bolts can adsorb magnetic particles to visualize magnetic marks. It has high sensitivity and can be used to detect cracks with micron width, and the display method is intuitive. The commonly used magnetic particle testing methods include yoke method, coil method, direct electromagnetic method, and induced current method. The magnetic particle method needs to clean the whole bolt completely before surface inspection. The magnetic memory method can be used to point to the stress concentration position in ferromagnetic structure and can also be used to detect the cracks of bolts. However, there are two problems in the above nondestructive testing methods: one is the need to remove the bolts. For some bolts that are not easy to disassemble, it is easy to damage the bolts by force; the other is that the sensors or equipment are too large to realize on-line monitoring, especially for aerospace agencies.

Physical testing and chemical inspection show that there are three periods in bolt cracking: crack initiation, crack propagation, and rapid fracture [4]. The existence of the crack propagation makes it possible to detect the crack and provide a timely warning before the period of the rapid fracture. Nevertheless, developing a real-time monitoring technique for bolt cracking it is both challenging and significant, and may provide essential data for assessing the remaining bearing capacity of the bolt and avoiding the secondary damage of the structure.

Structural health monitoring (SHM) technologies make it possible to monitor and predict the structural performance of bolted joints in real-time [5]. Some SHM technologies, such as vibration [6], acoustic emission [7], ultrasonics [8,9,10], impedance [11,12], comparative vacuum monitoring [13], vision [14], etc., have been used for hole-edge damage detection of bolted joints. Besides, Xu et al. [15] proposed a modified time reversal method to extract the phase shift and signal amplitude of focalized wave packet in the reconstructed signal as tightness indices for monitoring bolt losing in a lap joint. He et al. [16] proposed a method of bolt loosening detection that may identify the location of the bolt loosening by using the change of the natural frequency of the structure. Qiu et al. [17] proposed an enhanced metal magnetic memory method to monitor the fracturing failure of bolted joints for early prediction. Goldfine et al. [18] designed the rosette-type meandering winding magnetometer (MWM) sensor and further used it to monitor the cracks of the joint structure. Rakow and Chang [19] proposed one type of eddy current-sensing film to form the intelligent bolt by using the flexible printed circuit technique. This eddy current sensing film solved the problem of detecting the fatigue crack of thick joints or multi-layer joints, and it was optimized by Sun and Qing [20,21,22] for quantitative monitoring of the crack size and position.

However, for the bolt fracturing failure, the most common structural failure in the accidents [3,23], there are few SHM technologies. In this paper, the SHM concept of the embedding eddy current sensing films [19,20,21,22] is introduced to monitor the process of the bolt fracturing failure.

The structure of this paper is established as follows. In Section 2, two configurations of the coil array of eddy current sensing film are proposed to quantify the crack parameters of the bolt, and the interaction between the crack and the eddy current/magnetic field is further investigated. In Section 3, the ability of eddy current sensing film to quantify the crack is experimentally verified. Finally, some conclusions are drawn in Section 4.

## 2. Design and Simulation Analysis of Eddy Current Sensing Film

The purpose of this paper is to develop an eddy current array sensing film that can quantitatively monitor the process of bolt cracking. According to Sun and Qing’s study [20,21], when the direction of the eddy current generated by the exciting coil is perpendicular to the direction of the crack in the monitored structure, there is a significant disturbance of the eddy current which can be sensed by the sensing coils. A similar concept may be introduced to monitor the bolt cracking. The configuration of the sensing film will be designed according to the crack features of the bolt cracking, and the interaction between the crack and the eddy current/magnetic field generated by the sensing film will be analyzed.

### 2.1. The Design of the Sensing Film with One-Dimensional Eddy Current Coil Array

The crack surface of the bolt cracking is perpendicular to the axial direction of the bolt, which indicates that if the eddy current is horizontal to the axial direction, the crack has an apparent disturbance on the eddy current so that the sensing film has an excellent ability to track the bolt cracking.

**Figure 1:**
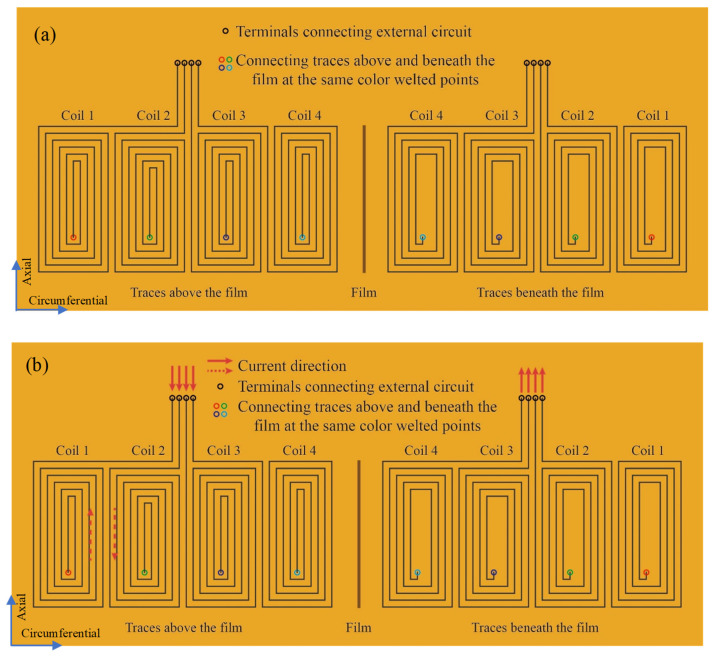
The configuration of the sensing film with one-dimensional coil array. (**a**) The configuration of sensing coil array; (**b**) The configuration of exciting coil.

First, the configuration of the sensing coil is designed. A one-dimensional sensing coil array in the circumferential direction is designed, and its parameters can be determined by specific actual monitoring requirements. For example, in Figure 1a, four rectangle coils distribute around the bolt, and each coil is made up of two portions printed above and beneath the film, respectively, which are connected at the middle of each coil. If a crack occurs at the area of one sensing coil, the signal of this sensing coil will change accordingly.

As shown in Figure 1b, the exciting coil has a similar configuration with the sensing coil, and the difference is that all the small coils are connected in series as one exciting coil, which means that at any location, the current is equal and the sensitivity of crack detection is the same. As the current is directional, the method of connecting in series affects whether the current directions of traces between adjacent coils are the same or opposite, which may cause the difference of the eddy current field. But the opposite current direction is helpful to split the eddy current field in the circumferential direction, which makes that it is easy to identify the crack angle.

### 2.2. Finite Element Simulation Model

Ansoft Maxwell, a commercial finite element software, is utilized to study the interaction between the crack and the eddy current/magnetic field.

**Figure 2:**
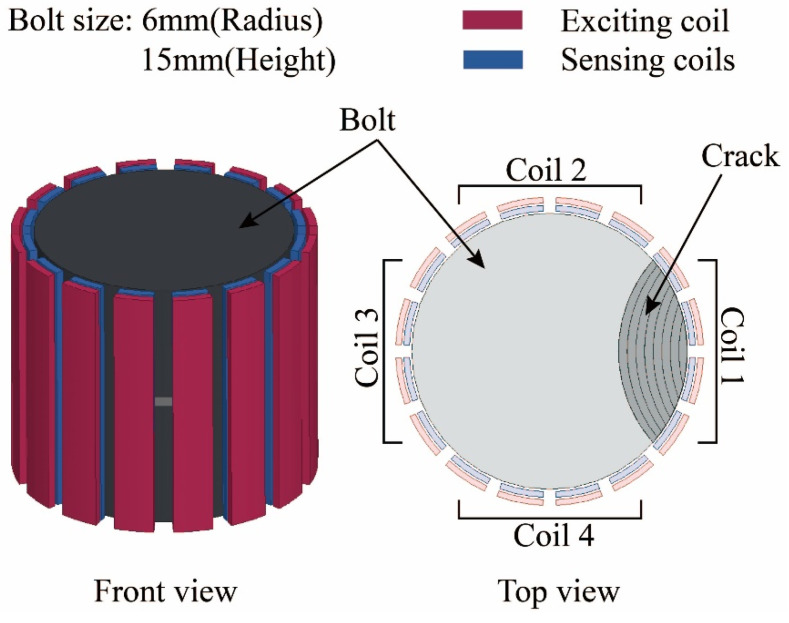
Finite element model.

As only bolt cracking is considered to influence the eddy current/magnetic field, the jointed structure has no change and is not included. The finite element model is shown in Figure 2. The bolt has a dimension of 15 mm (height) × 6 mm (radius), conductivity of 10,300,000 S/m, and relative magnetic permeability of 4000. Sixteen turns of traces are divided into four sensing coils, each with four turns. In contrast, another sixteen turns of traces are connected in series as the exciting coil. The trace width and spacing of the coil are both set as 0.1 mm. The lift-off distance between the sensing coil and the bolt surface is 0.1 mm. A sinusoidal signal with amplitude 1 V and frequency 100 kHz is used as the excitation voltage.

### 2.3. Quantitative Monitoring of the Bolt Cracking

The effect of the crack on the induced voltages of the sensing coils is explored. In Figure 2, the crack propagates from the middle of Coil 1 and expands in a step of 0.375 mm. The area covered by Coil 1 is analyzed, and the maximum depth of the crack is 3 mm. As the bolt and the coils are axisymmetric, it has the same effect on monitoring the bolt cracking when the crack is at the location of three other sensing coils. The effect of the crack on the induced voltage is marked as ΔV, which is calculated by the following formula,

(1) $$\Delta V=V_{m}-V_{0}$$

where $V_{m}$ and $V_{0}$ are the induced voltages of sensing coils with and without crack, respectively.

**Figure 3:**
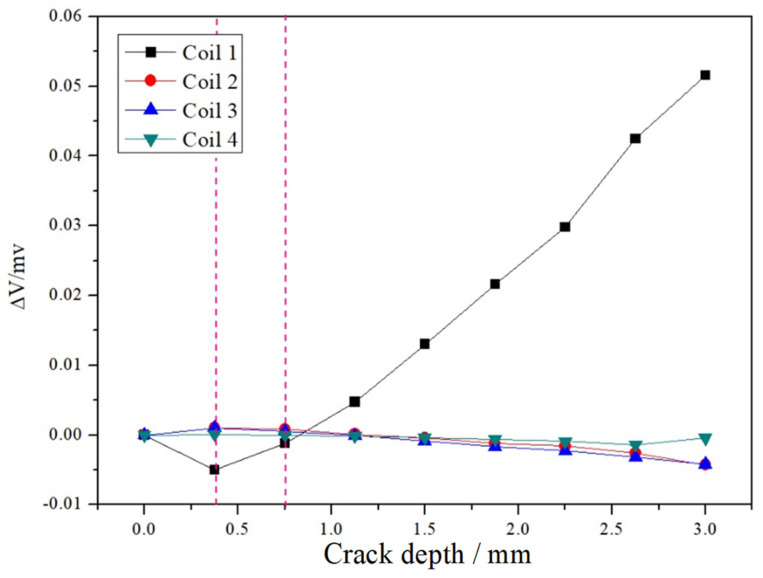
The change of induced voltage of sensing coils versus the crack propagation.

Figure 3 shows the variation of the induced voltage with the crack propagation. When the crack is initially set at the site of Coil 1, the induced voltage acquired by Coil 1 changes immediately, and those of the other sensing coils have no change. A noteworthy phenomenon is that the varying trend of the induced voltage of Coil 1 is not monotonous as the crack propagates, which needs further analysis.

For ferromagnetic materials, two main parameters affect the results of eddy current testing: conductivity and permeability [24]. The conductivity affects the eddy current field generated in the structure on the surface of and inside the bolt. If a crack is present, the crack impedes the flow of the eddy current, which will affect the induced voltage of the sensing coils. The magnetic permeability affects the binding of the original magnetic field. If the crack is present, the crack will destroy the binding effect of the bolt on the magnetic field and cause magnetic flux leakage, which reduces the intensity of the original magnetic field and further affects the induced voltage of sensing coils. In this paper, the variation tendency of the induced voltage of Coil 1 in Figure 3 can be analyzed from both the eddy current effect and magnetic flux leakage.

**Figure 4:**
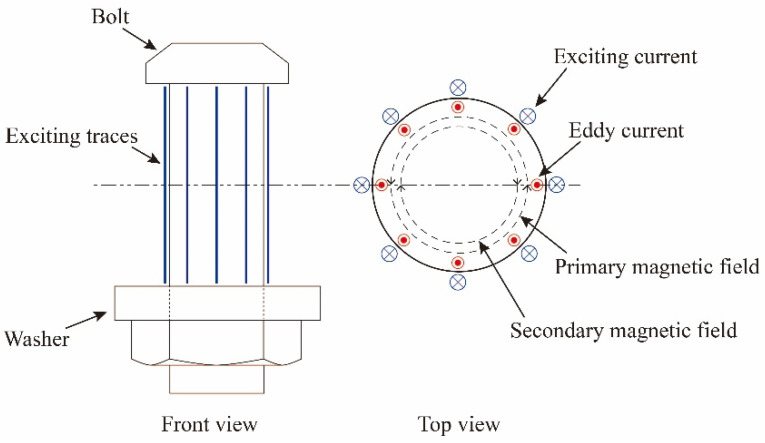
Schematic of eddy current effect.

First, the eddy current effect is studied. As shown in Figure 4, the traces of the exciting coil distribute around the bolt, and their current directions are in the axial direction of the bolt. The exciting current excites the alternating magnetic field in the counterclockwise direction, which generates an eddy current on the surface of the bolt with the opposite direction of the exciting current. The eddy current simultaneously excites a secondary magnetic field in a clockwise direction, which has an opposite phase with the original alternating magnetic field and weakens the original alternating magnetic field. If the crack occurs, the eddy current will be reduced. Then, the secondary magnetic field will decrease and the suppression of the original alternating magnetic field will be weakened. The spatial magnetic field around the sensing coils will be increased equivalently, which result in the rise of the induced voltage of the sensing coils.

**Figure 5:**
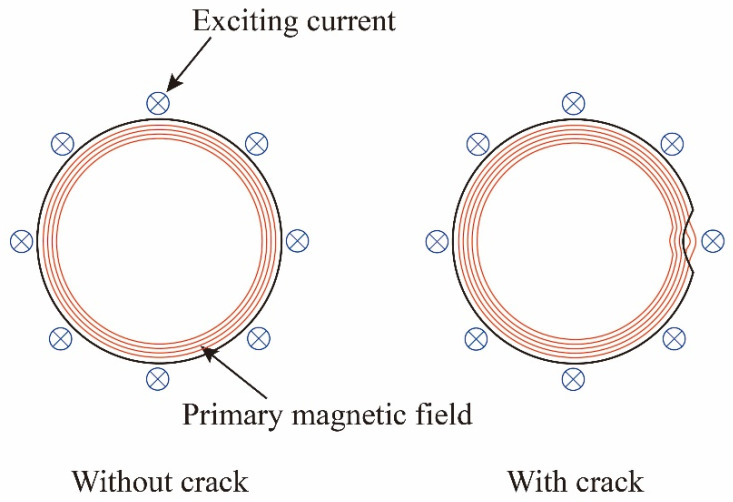
Schematic of magnetic flux leakage.

Second, the magnetic flux leakage is studied. The left part of Figure 5 shows the internal magnetic field distribution without the crack, which is excited by the exciting current. As the bolt is a ferromagnetic material, it has a high magnetic permeability and has a magnetic focusing effect on the magnetic field, that is, the magnetic field is confined on the surface of the bolt and the near-surface. When a crack initially occurs on the bolt, one part of the magnetic field at the crack leaks into the air, causing magnetic leakage, and the other part is forced to move in the crack surface of the bolt, as shown in the right part of Figure 5. It can be seen that the initiation of the crack decreases the ability of the bolt to restrain the magnetic field, which means the intensity of the magnetic field around the coil decreases, and the induced voltage of the sensing coil increases.

**Figure 6:**
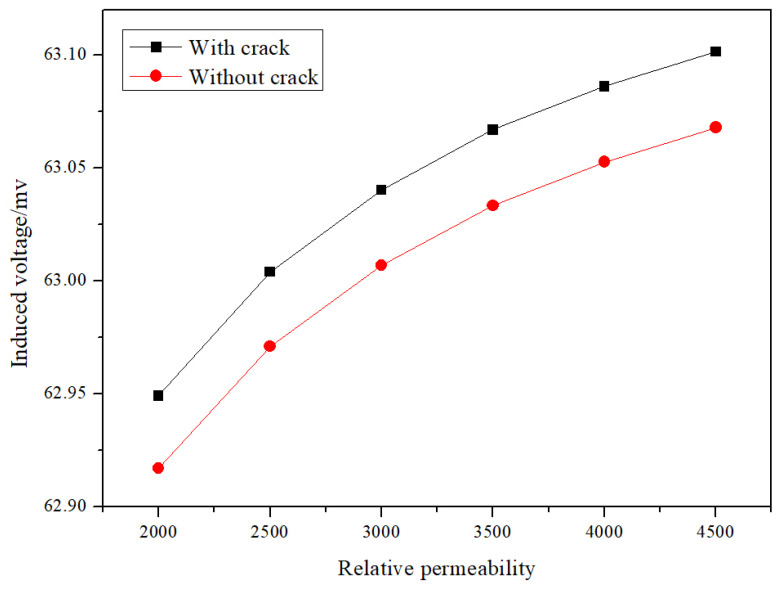
The variation of induced voltage of Coil 1 versus the relative permeability.

The magnetic flux leakage can be verified by two sets of simulation analysis. In the first simulation, different values of magnetic permeability are set for the bolts. The changes in the induced voltage of Coil 1 with and without crack under different magnetic permeability are shown in Figure 6. It can be seen that as the magnetic permeability increases, both the induced voltage of sensing coils with and without crack increase. As the magnetic permeability is proportional to the ability of the ferromagnetic material to bind the magnetic field, it can be deduced that when the ferromagnetic material is broken, the binding effect on the magnetic field reduces, and the induced voltage of the sensing coil decreases.

**Figure 7:**
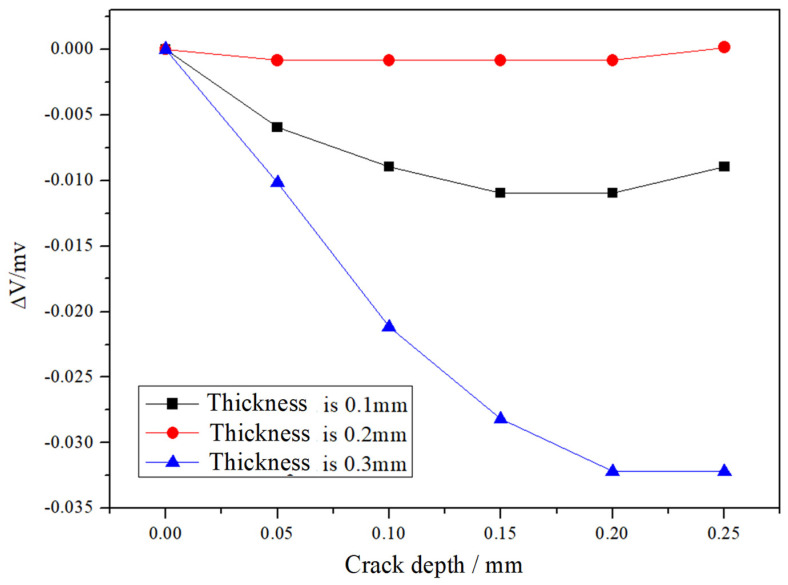
The variation of induced voltage of Coil 1 under different crack thicknesses.

In the second simulation, when the crack thickness is set as 0.1 mm, 0.2 mm, and 0.3 mm, respectively, the variations of the induced voltages of Coil 1 with the crack propagation are compared with each other. The greater the crack thickness, the greater the influence of the damage on the magnetic field of the ferromagnetic material when the crack is initiated. As shown in Figure 7, the induced voltage almost has no decrease when the crack thickness is 0.1 mm. When the crack thickness increases to 0.2 mm and 0.3 mm, the induced voltage has a much more significant reduction. It indicates that when the crack destroys the binding effect of the ferromagnetic material on the magnetic field, the induced voltage will decrease.

Based on the above analysis, the change in the induced voltage of Coil 1 in Figure 3 is divided into three stages: During Stage I, the crack initiates, and its depth is small, the influence of magnetic flux leakage is stronger than the eddy current effect, which causes a decrease of the induced voltage. In Stage II, the magnetic flux leakage is weakened as the crack propagates, and the eddy current effect is stronger than the magnetic flux leakage. It causes an increase in the induced voltage, but the voltage under this condition is still lower than that without crack. As the crack continues to propagate, it will progress to Stage III, where the induced voltage is large than that without crack and increases continuously as the crack propagates.

Although the varying tendency of the induced voltage with the crack propagation is not monotonous, Stages I and II are short so that they can be both considered in the period of the crack initiation. It is more critical to monitor Stage III, which can be considered in the period of the crack propagation.

It can be regarded as that the sensing film with one-dimensional coil array has an excellent ability for monitoring the angle of the bolt crack and the crack propagation.

### 2.4. Design and Verification of the Sensing Film with Two-Dimensional Coil Array

For multiple-lap bolted joints, the bending cracking may occur at an unknown axial location of the bolt. If more parameters are identified, the estimation of remaining life will be more accurate. In this section, the sensing film with a two-dimensional coil array is developed to get one more parameter of the bolt cracking, i.e., the axial location, than the above sensing film with the one-dimensional coil array.

**Figure 8:**
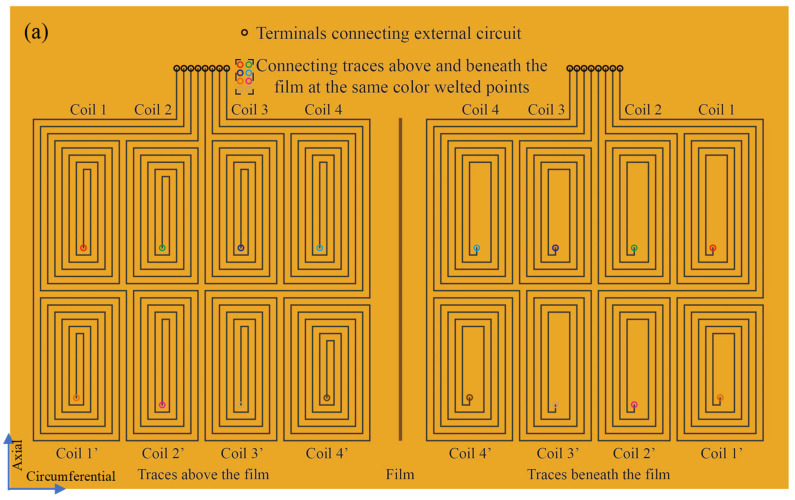

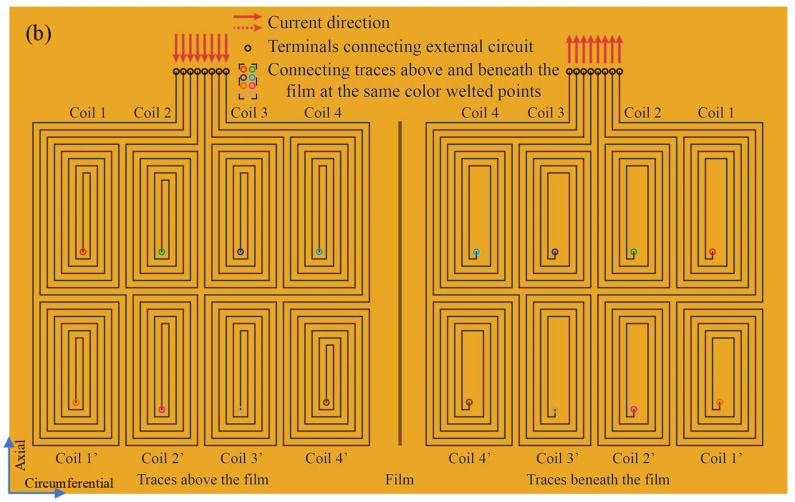
Sketch of the two-dimensional coil array sensing film. (**a**) Sketch of the sensing coil array; (**b**)Sketch of the exciting coil.

The configuration of the two-dimensional coil array sensing film is shown in Figure 8. Based on the above one-dimensional circumferential array, the two-dimensional coil array with 2 × 4 coils is introduced. If there are more coils in the axial direction, a more accurate axial location of the crack will be obtained. In the sensing layer, the induced voltages are acquired by eight sensing coils. In contrast, eight coils in the exciting layer are connected in series to one coil to ensure each trace with the same current.

**Figure 9:**
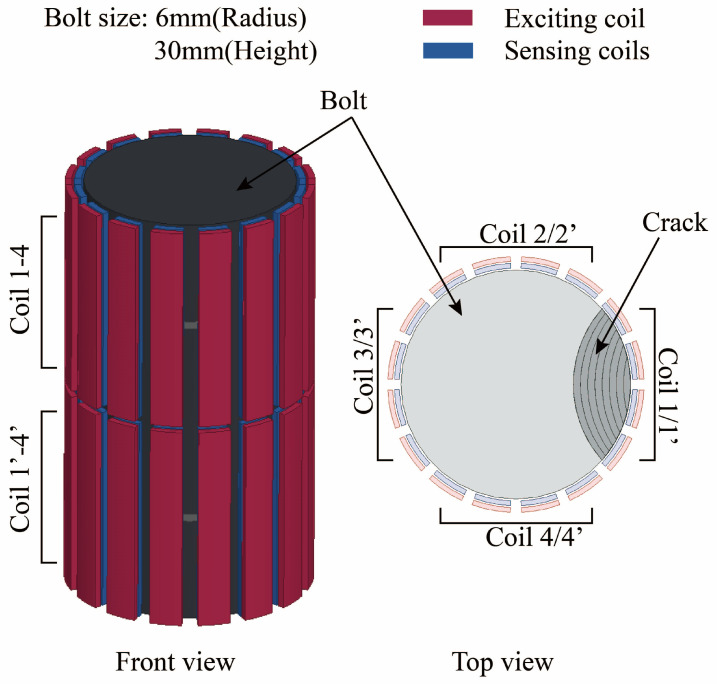
Finite element model of two-dimensional coil array sensing film.

The capability of the two-dimensional coil array sensing film is investigated in the simulation. Figure 9 gives the finite element model. Coils 1–4 and Coils 1′–4′ are distributed in the circumferential direction in the upper and lower halves of the bolt, respectively. Two cracks are set at the locations of Coil 1 and Coil 1′, respectively. The parameters of bolt material and crack are the same in Section 2.2.

**Figure 10:**
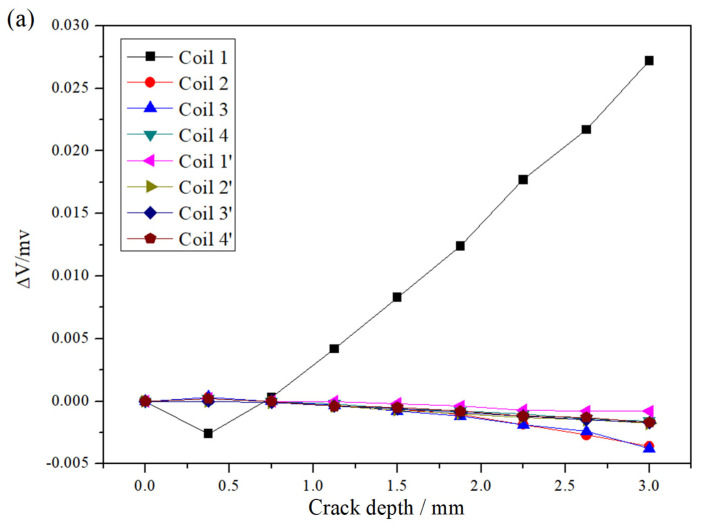

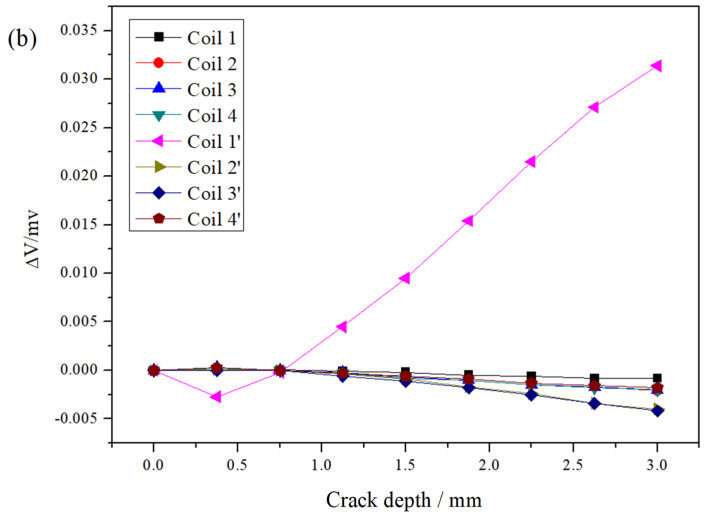
The variation of induced voltage versus the crack depth under different axial locations. (**a**) The crack at the site of Coil 1; (**b**) The crack at the site of Coil 1′.

The simulation results are shown in Figure 10. Figure 10a shows the variation of the induced voltages of eight sensing coils when the crack propagates at the site of Coil 1, while Figure 10b is the condition of the crack propagating at the site of Coil 1′. When the crack initiates and propagates at the site of one coil, the varying trend of the induced voltage of this coil is the same as in Section 2.3, while induced voltages of the other seven coils almost have no change.

It can be concluded that the proposed two-dimensional coil array sensing film can identify the axial location of the bolt cracking. Meanwhile, this sensing film retains the ability of a one-dimensional coil array sensing film to detect the crack angle and the crack depth.

## 3. Experimental Verification

The ability of the above two sensing films to monitor bolt cracking was verified by experiments. The eddy current sensing film has a dimension of 40.5 mm (length) × 18 mm (width), and its line width and space are both 0.1 mm. Each small coil has 30 turns. The sensing film was mounted on a steel screw with a hole diameter of 13 mm.

**Figure 11:**
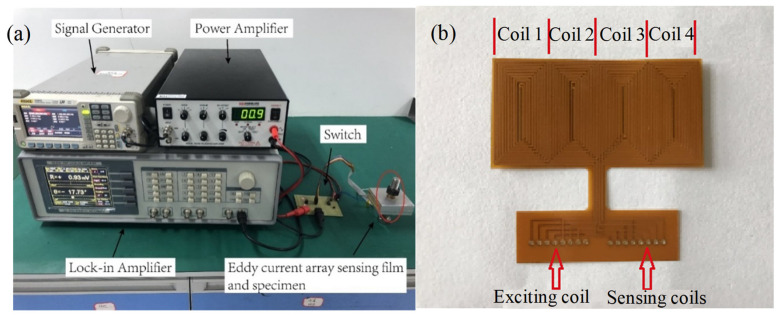
Experimental setup and sensor. (**a**) Experimental setup; (**b**) The sensing film.

The experimental system is given in Figure 11. The exciting signal of the exciting coil, with a frequency of 500 kHz, was generated by a signal generator Rigol DG3061A and amplified by a power amplifier Krohn-Hite 7600 M. The output signals of sensing coils were acquired by a lock-in amplifier OE2041 and a self-made switch.

**Figure 12:**
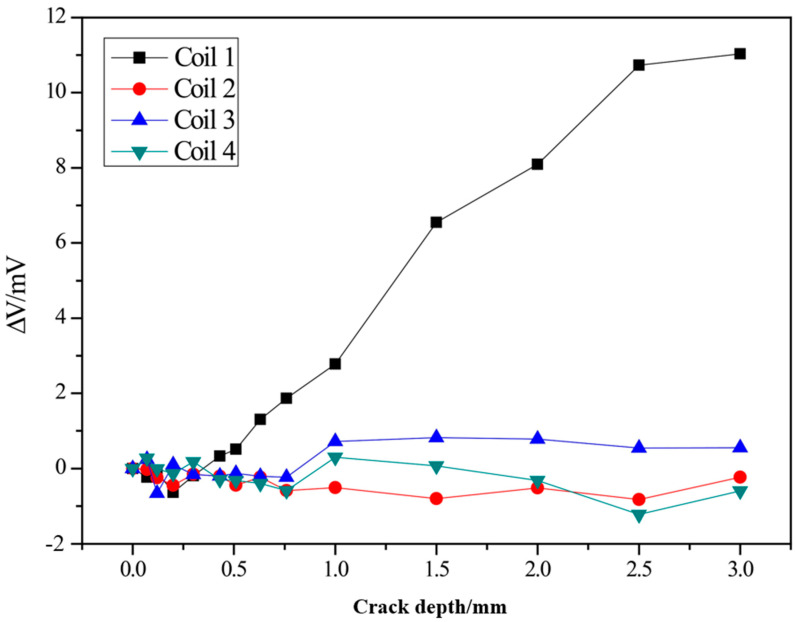
The experimental result of one-dimensional coil array sensing film.

The first experiment was used to check the ability of the one-dimensional coil array sensing film to monitor bolt cracking. The hole-edge crack is made by a laser cutting machine. To obtain the varying trend of induced voltage when the crack is initiated, the size of crack propagation follows the principle of small first and then big. The crack depth was set as 0.07 mm, 0.12 mm, 0.2 mm, 0.3 mm, 0.43 mm, 0.51 mm, 0.63 mm, 0.76 mm, 1.0 mm, 1.5 mm, 2.0 mm, 2.5 mm, and 3 mm, respectively. Figure 12 gives the change of the induced voltages of four sensing coils. When the crack propagates, the induced voltage of Coil 1 first decreases and then increases, while those of the other coils have slight fluctuations due to noise or measuring error. It indicates that one-dimensional circumferential coil array sensing film can identify the angle of the bolt cracking and track the crack propagation.

**Figure 13:**
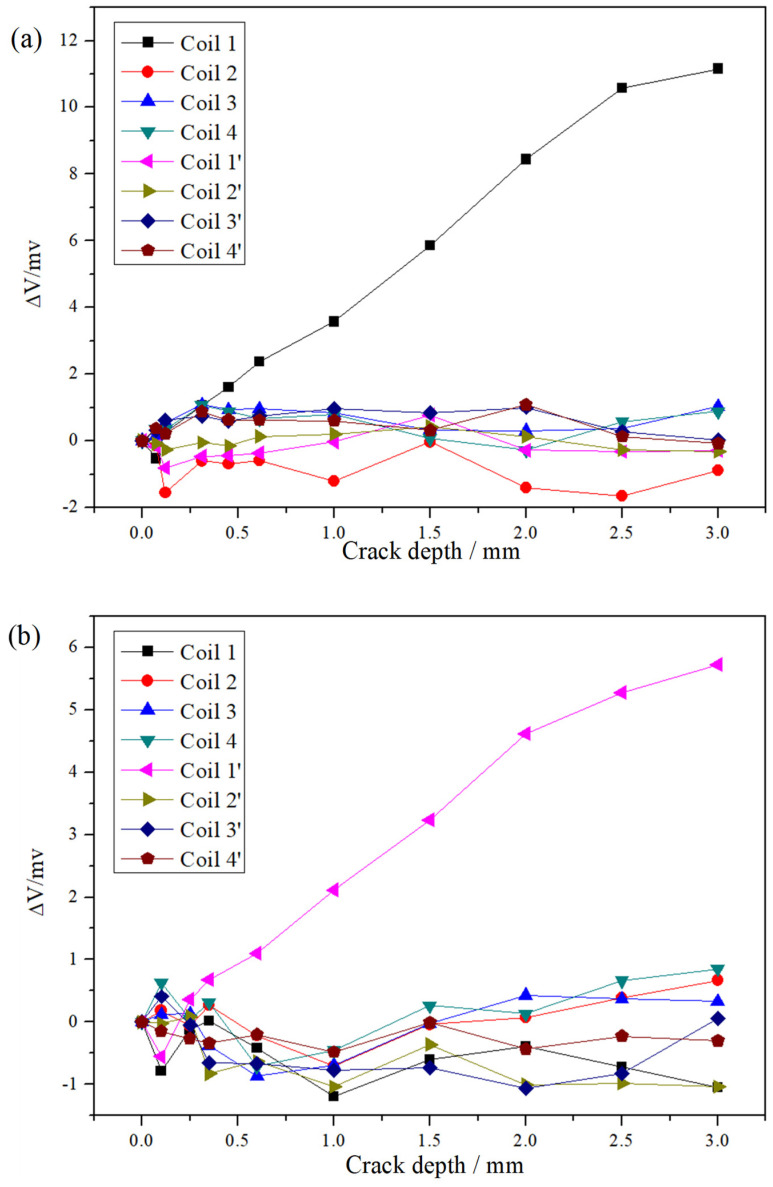
The experimental result of two-dimensional coil array sensing film. (**a**) The crack at the site of Coil 1; (**b**) The crack at the site of Coil 1′.

The second experiment was used to check the ability of the two-dimensional coil array sensing film to monitor the bolt cracking. The cracks were set at the middle of Coil1 and Coil1′, respectively. The crack depths were set as 0.07 mm, 0.12 mm, 0.31 mm, 0.45 mm, 0.61 mm, 1.0 mm, 1.5 mm, 2.0 mm, 2.5 mm, and 3 mm, respectively. Figure 13 shows the change of the induced voltages of eight sensing coils as the cracks propagate. When the crack occurs at the area of Coil 1, only the induced voltage of Coil 1 will change significantly, and the varying trend is the same as that of the one-dimensional coil array sensing film. The same phenomenon appeared when the crack was set at the site of Coil 1′. It indicates that the two-dimensional coil array sensing film has the capability of quantifying the axial location, the angle, and the depth of the bolt cracking.

The above two experiments prove that the proposed two types of eddy current sensing films can quantify the crack parameters of the bolt cracking.

## 4. Conclusions

In this paper, an eddy current sensing film has been proposed to quantify the crack on the bolt and estimate bolt cracking. Based on the finite element simulation and experiments, the ability of the sensing film has been verified. The conclusions can be made as follows.

**Figure 14:**
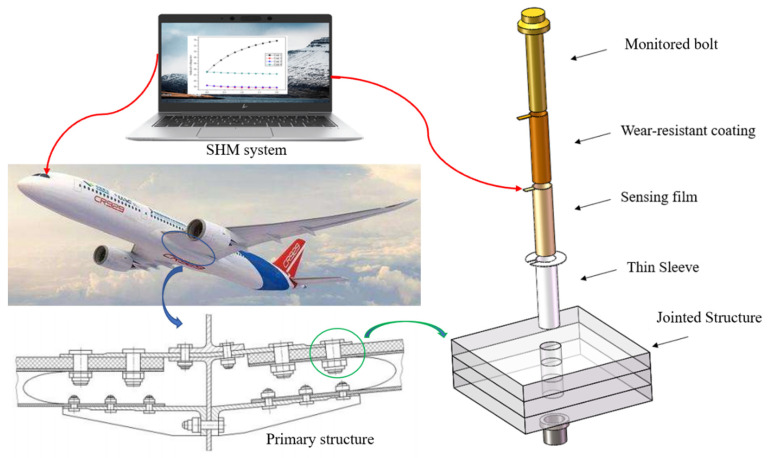
The structural health monitoring (SHM) system for monitoring the process of the bolt cracking.

(1) The one-dimensional circumferential array eddy current sensing film can identify the crack angle and the crack propagation on the bolt.
(2) For multi-lap bolted joints, a two-dimensional array sensing film can be used to effectively identify the axial location of the crack on the bolt, in addition to the crack angle and the crack propagation.
(3) The effects of conductivity and permeability on the detection results of ferromagnetic materials during eddy current testing have been deeply investigated. Both eddy current effect and magnetic flux leakage affect the variation of the induced voltage caused by the bolt cracking. Magnetic flux leakage plays a major role at the early stage of the bolt cracking, while eddy current effect does as the crack continuously propagates.
(4) The exciting frequency was considered as 100 kHz in the simulation and 500 kHz in the experiment. However, the same varying trend of induced voltage with the increase of the crack depth can be obtained in the range 100 kHz to 1 MHz.
(5) In order to ensure the reliability and durability of the sensing film in engineering applications, as shown in Figure 14, the flexible sensor film can be coated with a wear-resistant layer of SiO_2_ and Ta_2_O_3_ by electron beam physical vapor deposition and bonded to the inner surface of the sleeve, which is used for the bolted joints together with the monitored structure.
